# Alarming of Severe Respiratory Failure Requiring ECMO Caused by the SARS-CoV-2 Omicron Variant

**DOI:** 10.31662/jmaj.2022-0049

**Published:** 2022-05-30

**Authors:** Shinichiro Ohshimo, Nobuaki Shime, Tatsutoshi Shimatani, Yusuke Okazaki, Mitsuaki Nishikimi, Momoko Asada, Kohei Ota, Yuji Fujino, Shinhiro Takeda, Japan ECMO network

**Affiliations:** 1 Department of Emergency and Critical Care Medicine, Graduate School of Biomedical and Health Sciences, Hiroshima University, Hiroshima, Japan; 2 Non-profit Organization Japan ECMO Network, Kawaguchi, Japan; 3 Department of Anesthesiology and Intensive Care, Osaka University Graduate School of Medicine, Suita, Japan; 4 Kawaguchi Cardiovascular and Respiratory Hospital, Kawaguchi, Japan

**Keywords:** coronavirus, acute respiratory failure, mechanical ventilation

A novel severe acute respiratory syndrome coronavirus 2 (SARS-CoV-2) variant (B.1.1.529) named Omicron variant, which originated in South Africa and spread throughout the world with remarkable infectivity. However, large cohort analysis of patients infected with the Omicron variant in South Africa demonstrated a lower probability of hospitalization compared with other variants (adjusted odds ratio [OR], 0.2; 95% confidence interval [CI], 0.1-0.3) ^(1)^. Additionally, compared with patients infected with the Delta variant (B.1.617.2), patients infected with the Omicron variant had a lower rate of critical disease (adjusted OR, 0.3; 95% CI, 0.2-0.5). However, South Africa has experienced repeated episodes of SARS-CoV-2 outbreaks, and more than 70% of South Africans are estimated to have anti-SARS-CoV-2 antibodies, either through natural infection or vaccination ^(2)^. Therefore, whether the Omicron variant can induce severe respiratory failure in other cohorts without sufficient antibody titers should be carefully evaluated.

Here, we report a patient with severe respiratory failure due to SARS-CoV-2 Omicron variant infection, who required veno-venous extracorporeal membrane oxygenation (V-V ECMO). The patient was a 69-year-old woman with a height and weight of 159 and 85 kg, respectively (body mass index of 33.6) who had a history of well-controlled hypertension. She had no history of smoking, diabetes, chronic kidney disease, emphysema, fibrotic diseases, or any other significant complications. She was vaccinated with two doses of BNT162 mRNA vaccine against SARS-CoV-2 6 months ago. A family member of the patient had coronavirus disease 2019 (COVID-19), and the patient also underwent polymerase chain reaction (PCR) testing and genomic analysis, which was positive for the Omicron mutation (B.1.1.529). The patient did not receive any antiviral treatment and was resting at home. She started to present dyspnea 6 days later and was admitted to an emergency hospital the next day. Chest radiograph and computed tomography (CT) showed extensive bilateral ground-glass shadows and overlapping internal infiltrates (Figure 1). Laboratory tests showed white blood cells 13,000/μL, d-dimer 2.5 μg/mL, ferritin 2,505 ng/mL, KL-6 898 U/mL, CRP 22.0 mg/dL, and creatinine 2.7 mg/dL. The possibility of pulmonary embolism, cardiac failure, or concurrent infection was carefully excluded. Her SpO_2_ was 70% and her respiratory rate was 30 breaths/min (ROX index ^(3)^ of 3.89) under high-flow nasal cannula oxygen therapy with a fraction of inspiratory oxygen (F_I_O_2_) of 0.6 and prone positioning. She was therefore started on ventilatory management with tracheal intubation and prone positioning under muscle relaxation. However, 6 hours later, the ventilator settings were maximized as pressure-controlled ventilation mode with an F_I_O_2_ of 1.0, peak inspiratory pressure of 40 cmH_2_O, and positive end-expiratory pressure (PEEP) of 16 cmH_2_O to keep arterial partial pressure of oxygen (PaO_2_) of 60 mmHg. Murray’s score was 3.8, and the predicted mortality rate was over 80%. She was transported to our ECMO center and was started on V-V ECMO (Cardiohelp, Getinge AB, Göteborg, Sweden) using a 24 Fr drainage cannula via a right femoral vein (PCKC-V, Mera Development Corp., Tokyo, Japan) and an 18 Fr perfusion cannula via a right internal jugular vein (PCKC-A, Mera Development Corp., Tokyo, Japan) with blood flow of 3,500 L/min (2,800 rpm) and the sweep gas flow of 3.5 L/min. Currently, the patient is still on respiratory support using ECMO.

**Figure 1.**
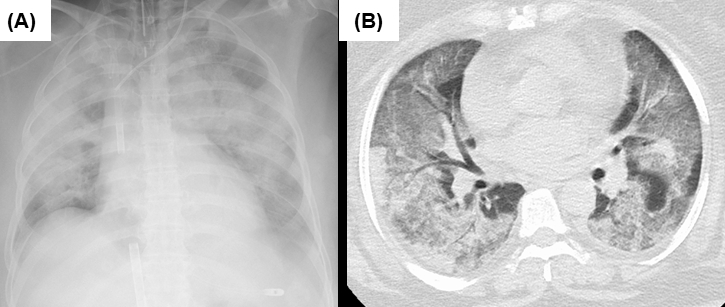
Chest radiograph and computed tomography on admission. (A) Chest radiograph right after cannulation of ECMO, which showed extensive ground-glass and infiltrative shadows in bilateral lung fields. A drainage cannula was placed in the inferior vena cava and a return cannula was placed in the superior vena cava. (B) Chest computed tomography on admission showed extensive ground-glass shadows in both lungs with partially overlapping infiltrative shadows. Interlobular septal wall thickening and traction bronchiectasis are unremarkable.

It has been widely recognized that the Omicron variant of SARS-CoV-2 was less likely to cause severe respiratory failure ^(1)^. However, this is the first reported case where the patient developed fatal respiratory failure requiring ECMO due to infection with the SARS-CoV-2 Omicron variant. Patients who have developed severe respiratory failure due to Omicron variant appear to be more likely to be unvaccinated, elderly, and have severe underlying diseases. In patients like our patient, who had been vaccinated and had no serious underlying diseases, Omicron infection rarely resulted in fatal respiratory failure requiring ECMO. According to a COVID-19 web database in Japan (CRISIS), the number of patients with severe respiratory failure with confirmed Omicron infection who are using ECMO appears to be limited ^(4)^. Chest CT demonstrated typical findings of the ARDS due to COVID-19, similar to those of the predominant variant in the earlier epidemic period.

In conclusion, this report may serve as a warning to remind us that Omicron variant infections are not always mild, even if vaccinated. The characteristics of the Omicron variant may provide important insights for the establishment of a social system to prevent the collapse of health care.

## Article Information

### Conflicts of Interest

None

### Sources of Funding

This work was supported by a Japan Society for the Promotion of Science (JSPS) KAKENHI Grant (Numbers JP 20K08541, 20H03782) and the Japan Agency for Medical Research and Development (AMED).

### Acknowledgement

The work of the Japan ECMO network is in cooperation with the ECMO Project Committee of the Japanese Society of Respiratory Care Medicine, the ECMO Project Committee of the Japanese Society of Intensive Care Medicine, and the ECMO Network Special Committee of the Japanese Association for Acute Medicine.

### Author Contributions

SO directly treated patients, conceived of the manuscript, and drafted the manuscript; NS, YF, and ST supervised and revised the manuscript; TS, YO, MN, MA, and KO directly treated patients and revised the manuscript.

The members of Non-profit organization Japan ECMO Network: SO, NS, YF, and ST.

### Approval by Institutional Review Board (IRB)

This study was approved by the ethical committee in Hiroshima University with the approval number of E-1965. Consent for publication of this paper was obtained from the patient's nearest relative.
